# What evidence is there for intraoperative predictors of perioperative cardiac outcomes? A systematic review

**DOI:** 10.1186/2047-0525-2-14

**Published:** 2013-07-03

**Authors:** Bruce M Biccard, Reitze N Rodseth

**Affiliations:** 1 Perioperative Research Group, Department of Anaesthetics, Nelson R. Mandela School of Medicine, University of KwaZulu-Natal, Private Bag 7, Congella 4013, South Africa; 2 Department of Anaesthetics, Inkosi Albert Luthuli Central Hospital, Durban, South Africa; 3 Population Health Research Institute, Hamilton Health Sciences, Hamilton, ON, Canada

## Abstract

**Background:**

Patients undergo cardiac preoperative evaluation to identify those at risk of adverse perioperative cardiac events. The Revised Cardiac Risk index is commonly used for this task, although it is unable to accurately risk stratify in all patients. This may be partly a result of intraoperative events which significantly modify preoperative risk.

**Methods:**

We conducted a systematic review to identify independent intraoperative predictors of adverse cardiac events in patients undergoing non-cardiac surgery. Four databases (Ovid Healthstar 1966 to Jan 2012, Ovid Medline 1946 to 6 March 2012, EMBASE 1974 to March 05 2012 and The Cochrane Library to March 06 2012) were searched.

**Results:**

Fourteen eligible studies were identified. The need for intraoperative blood transfusion (odds ratio (OR), 2.3; 95% confidence interval (CI), 1.4-3.3), vascular surgery (OR, 2.3; 95% CI, 1.2-3.4) and emergent/urgent surgery (OR, 2.3; 95% CI, 1.1-3.5) were the only independent intraoperative risk predictors identified in more than study. Other independent intraoperative factors identified included a >20 mmHg fall in mean arterial blood pressure for > 60 min (OR, 3.0; 95% CI, 1.8-4.9), >30% increase in baseline systolic pressure (OR, 8.0; 95% CI, 1.3-50), tachycardia in the recovery room (>30 beats per min (bpm) from baseline for >5 min) (OR, 7; 95% CI, 1.9-26), new onset atrial fibrillation (OR, 6.6; 95% CI, 2.5-20), hypothermia (OR, 2.2; 95% CI, 1.1-5) and remote ischemic preconditioning (OR, 0.22; 95% CI, 0.07-0.67). Other markers of surgical complexity were not independently associated with postoperative adverse cardiac outcomes. None of these studies controlled for blood transfusion.

**Conclusions:**

Intraoperative events significantly increase the risk for postoperative cardiac complications, although only intraoperative blood transfusion has strong evidence supporting this finding. It is possible that modification of these intraoperative risk factors by anesthetists and surgeons may reduce postoperative cardiac events and improve outcome. The Vascular Events in Noncardiac Surgery Patients Cohort Evaluation (VISION) Study will add important information to understanding intraoperative risk factors for adverse cardiac events.

## Key points

•There is good evidence that intraoperative blood transfusion is associated with perioperative adverse cardiac events

•Blood transfusion may obscure important physiological risk factors as it is a stronger signal than most other risk factors

•The Vascular Events in Noncardiac Surgery Patients Cohort Evaluation (VISION) Study may be able to determine the role of physiological variables in the presence of blood transfusion

## Background

The Revised Cardiac Risk Index (RCRI) [1] has been adopted by the American College of Cardiology’s/American Heart Association’s (ACC/AHA) [2] and the European Society of Cardiology/European Society of Anaesthesiology’s (ESC/ESA) guidelines for preoperative cardiac risk assessment [3]. Unfortunately, the RCRI has limited clinical application in identifying patients at risk of major adverse cardiac events (MACE). It is useful at excluding patients at risk of MACE (through an absence of known cardiac risk factors) [4], but it does not perform adequately in identifying patients at risk of MACE [4,5].

This may be partly explained by the complexity of the pathophysiology of perioperative cardiac events [6] (that is, sympathetic activation, hypoxia, procoagulation, the stress response). Most of these factors play a dominant role during the intraoperative period (and hence after risk stratification with the RCRI). This is evident in the National Surgical Quality Improvement Program (NSQIP) where traditional preoperative cardiac risk factors, lost their significance once intraoperative risk factors were considered [7].

We tested this hypothesis in a recent meta-analysis [8] where we aimed to determine which preoperative cardiac risk factors remained predictive of adverse cardiac events when taking into account independent intraoperative risk factors. To be eligible for inclusion in the meta-analysis, studies had to have examined both pre- and intraoperative risk factors associated with adverse cardiac events, using multivariable regression. The only independent intraoperative predictor that we were able to reliably identify was the need for intraoperative blood transfusion. In retrospect, limiting the inclusion criteria to those studies which controlled for preoperative cardiac risk factors may have severely limited the data available, and excluded studies that could provide useful information on which intraoperative factors are independently associated with adverse postoperative cardiac outcomes. To address this limitation, we have now conducted a systematic review of all studies reporting on intraoperative predictors associated with postoperative cardiac complications in patients undergoing non-cardiac surgery.

## Methods

We conducted a systematic review to identify independent intraoperative predictors of postoperative cardiac complications in patients undergoing non-cardiac surgery. Using the PICOT (patient/intervention/comparison/outcome/time) question structure [9] we framed the research question as: ‘Which intraoperative risk factors during non-cardiac surgery have been independently associated with adverse cardiac complications during the perioperative time period?’ The Preferred Reporting Items for Systematic reviews and Meta-Analysis (PRISMA) guidelines were followed for this review [10]. We did not register the review protocol for this meta-analysis.

### Study endpoints

From each study we intended to extract data on postoperative cardiac complications which included cardiac death, cardiac arrest, myocardial infarction, and myocardial ischemia.

### Study identification and selection

On 7 March 2012, RR conducted a search of four databases (Ovid Healthstar 1966 to Jan 2012, Ovid Medline 1946 to 6 March 2012, EMBASE 1974 to 5 March 2012 and the Cochrane Library to 6 March 2012). The search terms included: (1) (risk stratification or risk prediction or risk assessment).mp; (2) (intraoperative or perioperative).mp; and (3) (complications).mp. The exclusions were (cardiac surgery or coronary artery bypass or CABG).mp. The search was limited to English language, human, and all adult. All reviews, letters, case reports, comment, editorials, and guidelines were removed. We combined this search with a filter to maximize the sensitivity and specificity of the search, developed by the Health Information Research Unit [11]. All duplicate publications were removed. The search strategy is shown in Appendix 1.

RR and BB independently screened citations, abstracted data, and assessed methodological quality, using a standardized data extraction sheet. Disagreements were resolved through consensus. Full papers for all relevant citations were retrieved for detailed evaluation. Where potential intraoperative predictors of postoperative adverse outcomes were identified but not reported, the authors of studies were contacted for further data.

### Data analysis

The quality of each study was assessed for completeness of follow-up, method of patient follow-up, blinding of outcome adjudicators, and factors entered into the multivariable analysis. Concordance of article extraction was determined using a kappa statistic.

Independent intraoperative predictors were defined as intraoperative predictors which were retained in a multivariable model of risk factors for adverse perioperative cardiac events. Using published data from all studies, we determined adjusted odds ratios (OR) for all outcomes. All reported hazard ratios and risk ratios were converted to ORs for the meta-analysis [8].

The meta-analysis was conducted using RevMan version 4.3 software (The Nordic Cochrane Centre, Kobehavn, Denmark). We determined the adjusted OR for each study which we then pooled using the Der Simonian and Laird random effects model [12]. We calculated an I^2^ value to assess heterogeneity, and defined an I^2^ value ≤ 25% as low [13]. Our *a priori* hypotheses to explain heterogeneity, that is, I^2^ value >25%, included inconsistencies in the definitions used for intraoperative risk factors. Heterogeneity between studies was assessed using univariate chi-square analysis. Pooled dichotomous outcomes were reported as ORs and 95% confidence intervals (CI).

## Results

**Figure 1:**
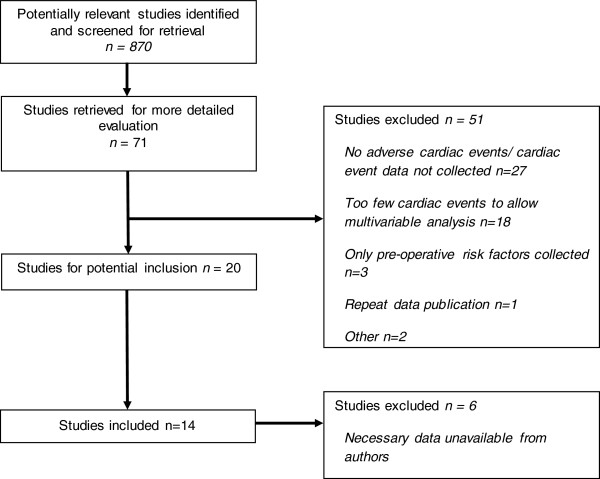
PRISMA flow diagram of study identification.

We identified 870 studies from the literature search, our own collections and through discussion with experts in the field. Seventy-one studies were identified for full paper analysis. The kappa statistic was 0.73. From these studies, 22 fulfilled our inclusion criteria, of which 14 were finally included (Figure 1) [7,14-27]. Seven papers were excluded as the authors could not be contacted or were unable to provide the data necessary for the analysis [28-34]. One further study [26] was excluded from this analysis as its data it was duplicated in a larger dataset [7,35].

The characteristics of the included studies are shown in Table 1. From the 15 included studies, eight were prospective [14,15,17,19,22,23,25,27]. These studies recruited patients from a variety of surgical disciplines, and included both elective and emergency patients. The outcome definitions varied between the studies.

**Table 1:** Characteristics and quality of studies included

**Author**	**Study design**	**Nature of surgery**	**Study outcomes**	**Follow-up length**	**Follow-up completeness (n/N)**	**Outcome assessment**	**Preoperative cardiac risk factors evaluated**
Charlson [14]	Observational: prospective	Cholecystectomy, vascular	Cardiac death, MI, myocardial ischemia	7 days postop or death or discharge	254/278 (91.4%)	Blinded	CAD, cardiomegaly (Only hypertensive and diabetic patients enrolled)
Ashton [23]	Observational: prospective and retrospective	Non-cardiac	MI	Hospital discharge	835/835 (100%)	Unblinded	Age, CAD, CF, cerebrovascular disease, chronic beta-blockade, diabetes, hypertension
Frank [15]	Prospective: randomized, single blind	Abdominal, thoracic, vascular	Cardiac arrest, MI, unstable angina	24 h postop	270/300 (90%)	Blinded	Age, ASA class, CAD, beta-blockade, diabetes, hypertension
Sprung [16]	Observational: retrospective	Vascular	Cardiac death, MI	Hospital discharge	6,948/6,948 (100%)	Unblinded	CAD, CF, beta-blockade, valvular heart disease
Ali [17]	Prospective: randomized, single blind	Vascular	MI, myocardial injury	7 days postop	82/82 (100%)	Blinded	Age, CAD, hypertension, NYHA functional capacity, POSSUM score
Davenport [36]	Observational: retrospective	General, vascular	Cardiac arrest, MI	30 days postop	182,900/183,069 (99.9%)	Unblinded	Age, ASA class, CF, CVA, gender, renal dysfunction
Bursi [18]	Observational: retrospective	Vascular	Death, MI	30 days postop	359/359 (100%)	Blinded	Age, CAD, diabetes, gender, hypertension, renal dysfunction, smoking history
Matyal [25]	Observational: prospective	Vascular	MI, CF, significant arrhythmia, prolonged intubation, renal failure, death	30 days postop	325/325 (100%)	Unblinded	Age, renal dysfunction
Oscarsson [19]	Observational: prospective	Non-cardiac	Cardiac death, MI	Hospital discharge	186/211 (88%)	Unblinded	Age, CF, diabetes, diuretics, nitrates
Winkel [20]	Observational: retrospective	Vascular	Cardiac death, MI, unstable angina	12-48 h postop & 30 days to 3 months postop	317/317 (100%)	Unblinded	Cardiac risk classification, gender, hypertension
D’Ayala [21]	Observational: retrospective	Lower limb amputations	MI, postop cardiac dysrhythmia	30 days postop	300/300 (100%)	Unblinded	CAD, diabetes, hypertension, renal dysfunction
Martin [24]	Observational: retrospective	Vascular	Death, MI	Hospital discharge	403,974/403,974 (100%)	Unblinded	Age, CF, diabetes, renal dysfunction
Devereaux [22]	Prospective: randomized, controlled, blinded	Non-cardiac	Death, MI	Hospital discharge	8,331/8,351 (99.7%)	Unblinded	Age, CF, heart rate, renal dysfunction, statin therapy
Rodseth [27]	Prospective: observational	Vascular	Death, troponin elevation	30 days postop	149/149	Unblinded	Preoperative B-type natriuretic peptides

*ASA* American Society of Anesthesiologists, *CAD* Coronary artery disease, *CF* Cardiac failure, *CVA* Cerebrovascular accident, *MI* Myocardial infarction, *NYHA* New York Heart Association.

### Independent intraoperative factors predicting postoperative cardiac complications

Ten intraoperative factors were identified as independent predictors of adverse postoperative cardiac events. We classified these as surgical risk factors (that is, surgery complexity, urgency, and the requirement for blood transfusion), physiological risk factors (that is, tachycardia, hypotension, hypertension, hypothermia, and diastolic dysfunction) and an interventional predictor (remote ischemic preconditioning).

### Blood transfusion

**Figure 2:**
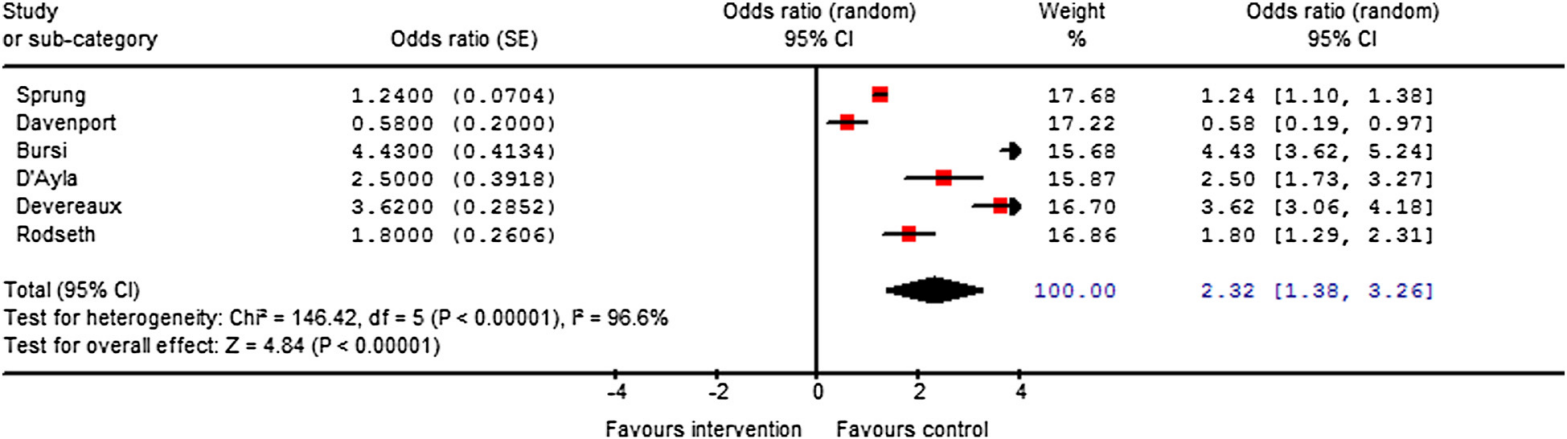
Meta-analysis of peri-operative blood transfusion and associated adverse cardiac events

Perioperative blood transfusion was identified in six studies as the risk factor most commonly identified as being independently associated with adverse postoperative cardiac outcomes [7,16,18,21,22,27]. Intraoperative blood transfusion was associated with significantly increased adverse cardiac events (OR, 2.3; 95% CI, 1.4-3.3) (Figure 2). There is significant heterogeneity (I^2^=96.6%) in this point estimate, probably due to variations in study definitions of postoperative cardiac complications and blood transfusion. These definitions include bleeding disorder [7], blood given [21], units of blood given [16,27], and serious bleeding, defined as disabling bleeding or two or more units given [22].

### Surgical complexity

Surgical complexity (as defined by the duration of surgery or by categorization of the complexity of the procedure) was inconsistently associated with adverse cardiac outcomes. Two studies controlled for the duration of surgery [7,27]. However, when blood transfusion was entered into a multivariable analysis of postoperative cardiac outcomes, duration of surgery did not remain independently associated with adverse outcomes [7,27].

Using work relative value units (RVU), a measure developed by the Center for Medicare in the USA to classify surgical complexity [7], the authors showed that surgery with a RVU >17 as compared to <10 was associated with significantly increased adverse postoperative cardiac outcomes (OR, 3.0; 95% CI 2.3-3.8) [7]. Surgical complexity was not confirmed in the second study which controlled for this variable in a study of aortic vascular surgery [24]. However in this study, surgical complexity was associated with greater mortality following perioperative myocardial infarction, when patients who had a visceral resection in addition to aortic surgery were compared to patients who had aortic surgery alone (OR, 6; 95% CI, 5.3-6.9; *P* < 0.001) [24].

### Surgical urgency

**Figure 3:**
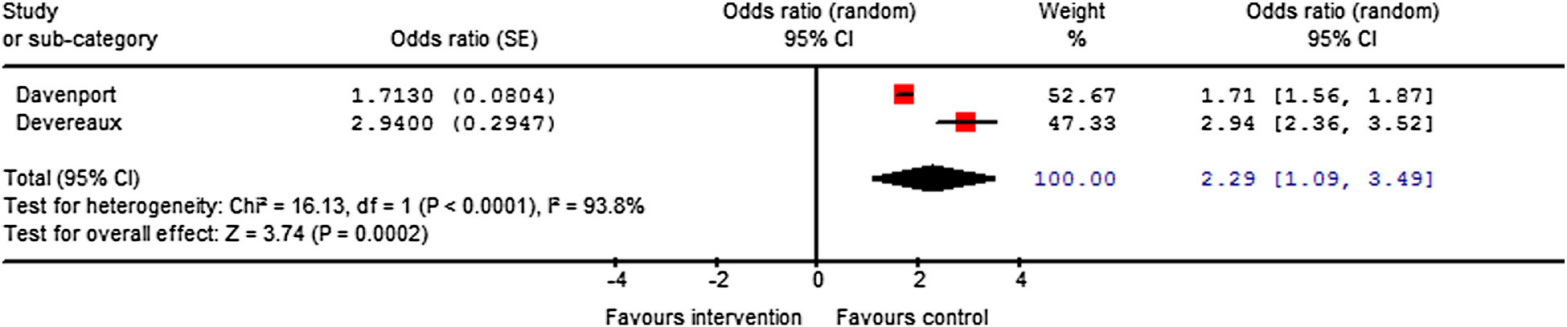
Meta-analysis of urgent surgery and associated adverse cardiac events.

Two publications reported on the association between urgent or emergency surgery and adverse cardiac outcomes [7,22]. The random effects model for urgent or emergency surgery was associated with increased adverse cardiac outcomes (OR, 2.3; 95% CI, 1.1-3.5; *P *= 0.0002), but with significant heterogeneity (I^2^= 93.8%) (Figure 3).

### Vascular surgery

**Figure 4:**
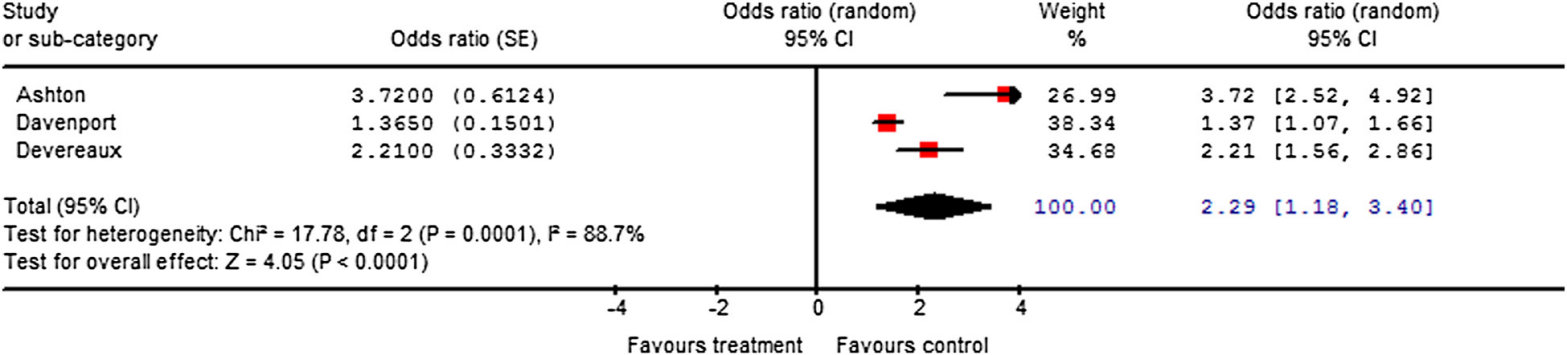
Meta-analysis of vascular surgery and associated adverse cardiac events.

Three studies found that vascular surgery, when compared to other forms of noncardiac surgery, was independently associated with adverse postoperative cardiac outcomes (OR, 2.3; 95% CI, 1.2-3.4; *P *= 0.0001; I^2^= 88.7%) (Figure 4) [7,22,23].

### Physiological variables

Cardiovascular physiological variables were also identified as being independently associated with postoperative cardiac adverse events. These included a >20 mmHg fall in mean arterial blood pressure for > 60 min (OR, 3.0; 95% CI, 1.8-4.9) [14], >30% increase in baseline systolic pressure (OR, 8.0; 95% CI, 1.3-50) [19], tachycardia in the recovery room (that is, >30 beats per min (bpm) from baseline for > 5 min (OR, 7; 95% CI, 1.9-26)) [19], and transmitral flow propagation < 45 cm.s^-1^ (OR, 25; 95% CI, 1.3-4.6) [25]. However, in the only study which controlled for blood transfusion, hypotension and tachycardia were no longer independently associated with adverse cardiac events [26].

### Other intraoperative risk factors

Other factors independently associated with increased adverse cardiac events included new onset atrial fibrillation (OR, 6.6; 95% CI, 2.5-20) [20] and hypothermia (OR, 2.2; 95% CI, 1.1-5) [15]. Remote ischemic preconditioning (OR, 0.22; 95% CI, 0.07-0.67) [17] was associated with cardioprotection. None of these studies controlled for blood transfusion.

## Discussion

This systematic review suggests that perioperative blood transfusion, vascular surgery, and urgent/emergent surgery are independently associated with postoperative cardiac events. Some studies suggest an association between changes in intraoperative hemodynamic variables and adverse cardiac outcome, although these data are insufficient for meta-analysis, and most do not control for intraoperative blood transfusion.

The high-risk nature of both vascular and urgent/emergent surgery have been well recognized [2,3] and are largely unmodifiable. This review confirms the strong association between intraoperative blood transfusion and adverse postoperative cardiac events after non-cardiac surgery. Perioperative transfusion should flag patients as particularly high risk for adverse cardiac events. Unfortunately the varying study definitions of exposure and outcomes mean that we are unable to determine an accurate point estimate associated with this risk. However, the need for blood transfusion may represent a significant multifaceted physiological insult, in which hypotension, tachycardia, hypothermia, and anemia may all contribute to the development of adverse cardiac outcomes. It is not surprising then, that hemodynamic variables are no longer predictive when considered together with the need for blood transfusion [26].

It is possible, that in the absence of the need for perioperative blood transfusion, physiological variables are associated with adverse cardiac outcomes. However, this review highlights the paucity of data reporting the impact of physiological variables on postoperative cardiac outcomes. Studies identified in this review suggest that prolonged hypotension, hypertension, tachycardia, and hypothermia may be associated with adverse postoperative cardiac outcomes. These risk factors are potentially modifiable by perioperative physicians and may present opportunities to improve patient outcomes. It is imperative that the association between potentially undesirable physiological factors and adverse cardiac outcomes are further delineated in a large observational study. Furthermore, when attempting to identify independent intraoperative predictors of adverse cardiac outcomes, future investigators should also control for preoperative cardiac risk factors.

In one of the seven studies which assessed the need for blood transfusion on adverse cardiac outcomes [27], preoperative B-type natriuretic peptide risk stratification was significantly improved upon when intraoperative blood transfusion was considered. This is an important observation, as there is high-level evidence that BNP is significantly better than the RCRI at preoperative risk stratification for adverse cardiac events in vascular surgical patients [37,38]. Future studies should also attempt to include this preoperative risk factor in their analysis.

In order to control for both preoperative and intraoperative cardiac risk factors, future studies will require much larger sample sizes. This meta-analysis identified 12 possible pre- and intraoperative cardiac risk factors: the RCRI criteria, urgency of surgery, intraoperative tachycardia, hypo and hypertension, hypothermia, and blood transfusion. To evaluate these variables in a population with an adverse event rate of 5% [22], and with a ratio of 12 events per variable [39], a sample size of 2,880 would be required. It is also important to determine from a subcohort of patients who did not require a blood transfusion, which intraoperative physiological variables are independently associated with adverse cardiac outcomes in the presence of established preoperative cardiac risk factors, as this may identify important physiological intervention thresholds for anesthetists.

Fortunately a study of this description; the Vascular Events in Noncardiac Surgery Patients Cohort Evaluation (VISION) Study is currently underway [40]. The VISION Study is an international, prospective, observational study of non-cardiac surgery patients. One of the main aims of the study is to determine the pathophysiology of cardiovascular events and develop optimal models for predicting perioperative mortality and morbidity.

In the presence of the preoperative VISION model, a postoperative troponin leak can explain over 40% of the population attributable risk for mortality at 30 days [40]. Therefore postoperative troponins are a very strong objective marker of adverse cardiac outcomes. Work is currently underway to determine an optimal intraoperative model for MACE using the VISION data. With a sample size of over 12,000 patients, it is possible to introduce all the potentially important preoperative and intraoperative risk factors into a logistic regression. The results from VISION should vastly improve our understanding which intraoperative risk factors are associated with adverse cardiac outcomes. These data will provide useful information necessary for designing perioperative intervention trials to improve patient outcome following non-cardiac surgery.

## Conclusions

There is a paucity of data concerning intraoperative predictors of MACE following non-cardiac surgery. Intraoperative predictors may be related to surgical complexity and physiological insult. Urgent non-cardiac surgery and vascular surgery are known to increase postoperative cardiac events. Additionally, there is strong evidence to suggest that patients who receive blood transfusions in the intraoperative period have worse cardiac outcomes than those who are not transfused. However, further research is required to understand the relationship between preoperative and intraoperative risk factors (including both surgical risk factors and physiological insults) and adverse cardiac outcomes. In this regard the analysis of the VISION data is eagerly awaited.

## Appendix 1. Search strategy and databases

Database searches were conducted on 7 March 2012 using the OvidSP search engine (Ovid Technologies, Inc., New York, NY 2009) for the following databases:

1. EMBASE 1974 to 5 March 2012

2. OVID Health Star (1966 to January 2012)

3. Ovid MEDLINE(R) In-Process & Other Non-Indexed Citations and OVID MEDLINE(R) 1946 to 6 March 2012

4. Cochrane Central Register of Controlled Trials (6 March 2012)

5. Cochrane Database of Systematic Reviews (6 March 2012)

Example of search conducted on MEDLINE

Search terms

1. (risk stratification or risk prediction or risk assessment).mp.

2. (intraoperative or perioperative).mp

3. Complications.mp

4. (cardiac surgery or coronary artery bypass or CABG).mp.

5. 1 AND 2 AND 3

6. 5 NOT 4

7. Limit to English language, human, all adult

8. NOT: randomized clinical trials, review, letters, case reports, comment, editorial, guideline

9. remove duplicates from 8

## Abbreviations

ACC/AHA: American College of Cardiology/American Heart Association; CI: Confidence interval; ESC/ESA: European Society of Cardiology/European Society of Anaesthesiology; MACE: Major adverse cardiac events; NSQIP: National Surgical Quality Improvement Program; OR: Odds ratio; PICOT: (Patient/intervention/comparison/outcome/time); PRISMA: Preferred Reporting Items for Systematic reviews and Meta-Analysis; RCRI: Revised Cardiac Risk Index; RVU: Relative value units; VISION: Vascular Events in Noncardiac Surgery Patients Cohort Evaluation Study.

## Competing interests

Dr Biccard has no affiliation with industry and declares that he has no conflict of interest. Dr Rodseth has no affiliation with industry and declares that he has no conflict of interest.

## Authors’ contributions

Biccard made substantial contributions to conception and design, acquisition of data, and analysis and interpretation of data. He was involved in drafting the manuscript and gave final approval of the version to be published. Rodseth made substantial contributions to conception and design, acquisition of data, and analysis and interpretation of data. He was involved in critically revising the manuscript for important intellectual content and gave final approval of the version to be published.
